# “Statistics 103” for Multitarget Tracking

**DOI:** 10.3390/s19010202

**Published:** 2019-01-08

**Authors:** Ronald Mahler

**Affiliations:** Random Sets LLC, Eagan, MN 55122, USA; MahlerRonald@comcast.net

**Keywords:** multitarget tracking, random finite set, point process, finite-set statistics

## Abstract

The finite-set statistics (FISST) foundational approach to multitarget tracking and information fusion was introduced in the mid-1990s and extended in 2001. FISST was devised to be as “engineering-friendly” as possible by avoiding avoidable mathematical abstraction and complexity—and, especially, by avoiding measure theory and measure-theoretic point process (p.p.) theory. Recently, however, an allegedly more general theoretical foundation for multitarget tracking has been proposed. In it, the constituent components of FISST have been systematically replaced by mathematically more complicated concepts—and, especially, by the very measure theory and measure-theoretic p.p.’s that FISST eschews. It is shown that this proposed alternative is actually a mathematical paraphrase of part of FISST that does not correctly address the technical idiosyncrasies of the multitarget tracking application.

## 1. Introduction

The finite-set statistics (FISST) foundational approach to multitarget tracking and information fusion—stochastic geometry, random finite sets (RFS’s), belief-mass functions, and set derivatives and integrals—was introduced in the mid-1990s [1]. Its current extended form—probability generating functionals (p.g.fl.’s) and Volterra functional derivatives [2,3,4]—dates from 2001 [5]. FISST has inspired work by dozens of research groups in at least 20 nations; and FISST publications have been cited tens of thousands of times. A short survey of the FISST state-of-the-art c. 2015 can be found in Ref. [6]. The currently most advanced FISST-based algorithm, the generalized labeled multi-Bernoulli (GLMB) filter [3,7,8], is capable of real-time tracking of over one million 2D targets in clutter using off-the-shelf computing equipment [9].

FISST was devised to be as “engineering-friendly” as possible by *avoiding avoidable mathematical abstraction and complexity* [4]. Few tracking engineers have studied measure theory and far fewer are proficient. Still fewer have studied point process (p.p.) theory (which typically requires proficiency in measure theory), and few are proficient. For this reason, FISST does not employ measure theory or measure-theoretic p.p.’s, because simpler and more practical concepts, such as multitarget density functions, RFS’s, and Volterra functional derivatives, suffice.

Despite its “engineering-friendly” emphasis, FISST has inspired two rather contradictory reactions. Some have insinuated that FISST is probably unnecessary because it will probably turn out to be just a mathematical obfuscation of multi-hypothesis tracker (MHT) theory. Such a stance is quite mistaken and has been addressed in the tutorial [10].

Others, however, have recently intimated that FISST is *insufficiently complex* because it is *insufficiently general*. They have systematically replaced the constituent components of FISST with mathematically more complicated concepts—and, especially, with the very measure theory and measure-theoretic p.p. theory that FISST eschews.

It has been my observation, as well as that of others, that most tracking engineers—even those very familiar with measure and p.p. theories—must invest a great deal of effort to digest such papers in order to decipher their possible contributions. It is thus entirely possible for mathematical complexity to obscure mathematical or engineering missteps. A central question, therefore, is this: What *engineering* advantages, if any, do sigma-algebras, measures, measure-theoretic p.p.’s, and other mathematically sophisticated concepts offer—especially given that FISST-based algorithms such as the GLMB filter offer unprecedented capability?

This review paper, which is a sequel to Refs. [4,11], is intended to answer this question. It will address the alternative multitarget statistical theory described in Refs. [12,13,14], with (for the sake of specificity) emphasis on Ref. [13]. This theory will hereafter be referred to as “measure-theoretical point-process multitarget tracking”, or “MPMT” for short. It will be demonstrated that MPMT is *a mathematical paraphrase* of part of FISST—one that, moreover, does not correctly address the technical idiosyncrasies of the multitarget tracking application.

A mathematical paraphrase is a substitution of terminology, notation, concepts, ideas, or processes that are *equivalent in mathematical meaning* to the terminology, notation, concepts, ideas, or processes in the original. In particular, it will be shown that:

1. When applied to practical multitarget tracking, p.p.’s are not “more general” than RFS’s.
2. The “regional variance” of Ref. [12] *does* admit a density—thereby refuting the only offered evidence in Refs. [12,13,14] that MPMT is unavoidable for practical multitarget tracking.
3. When applied to multitarget tracking, the “chain differential” is identical to the Gâteaux and Frechét derivatives—and thus mathematically equivalent to the FISST functional derivative.

These, and other noteworthy facts that follow, have thus far been overlooked in the tracking literature. It is therefore important that such oversights be carefully addressed.

The paper will address the following replacements of FISST concepts with MPMT concepts: RFS’s with p.p.’s (Section 2); FISST densities with “measures” (Section 3); set integrals with measure-theoretic integrals (Section 4); functional derivatives with “chain differentials” (Section 5); the FISST product rule with “Leibniz’ Rule” (Section 6); and RFS motion models with p.p. motion models (Section 7). Mathematical derivations can be found in Section 8 and Conclusions in Section 9. The discussions have been made as tutorial as feasible.

## 2. RFS’s Replaced by “Point Processes” (p.p.’s)

MPMT replaces RFS’s with “…the more general concept of point process” [13] (p. 1324) (This phase is logically vacuous: “more general” than what? What is implicitly meant is “more general than RFS’s”.). Specifically, if ℜ denotes the real numbers then:

“…the population of targets is represented by a point process Φ, on a single-target state space **X** ⊆ ℜ*^d^*, whose elements describe individual target states. A realization of Φ is a vector of points *ϕ* = (*x*_1_, …, *x_N_*) depicting a specific multitarget configuration, where *x_i_* ∈ **X**… A point process Φ is characterized by its probability distribution P_Φ_ on the measurable space (X, **B**_X_), where X = ∪*_n_*_≥0_**X***^n^* is the point process state space, i.e., the space of all the finite vectors of points in **X**, and **B**_X_ is the Borel σ-algebra on X… The probability distribution of a point process is defined as a symmetric function, so that the order of points in a realization is irrelevant for statistical purposes…”.

[13] (p. 1325)

The following subsections address the topics: RFS’s are not an “alternative construction” (Section 2.1); RFS’s are simpler than simple p.p.’s (Section 2.2); non-RFS p.p.’s are inappropriate for multitarget tracking (Section 2.3); vectors are poor multitarget state representations (Section 2.4); simple p.p.’s produce a flawed mathematical paraphrase of RFS’s (Section 2.5); and FISST is actually more general than MPMT (Section 2.6).

### 2.1. RFS’s Are Not an “Alternative Construction”

In MPMT, p.p.’s are assumed to be “simple” (i.e., the *x*_1_, …, *x_n_* in (*x*_1_, …, *x_n_*) are distinct), while it is also asserted that an RFS is an “alternative construction” of a simple p.p. that is “also available in the literature” [13] (p. 1325, footnote). *This is misleading*. It is simple p.p.’s that are being proffered as an alternative to RFS’s *for application to multitarget tracking*.

It could be argued to the contrary that, in *the pure-mathematics literature of many decades ago*, RFS’s historically arose as an alternative to the three formulations of p.p.’s originally proposed by Moyal in Ref. [15]. However, any such claim overlooks the following fact: *When signal processing engineers apply concepts drawn from the pure-mathematics literature, they typically create original intellectual property which must be properly acknowledged as such* (Otherwise, why would one need signal processing engineers?) Moyal’s paper addressed no practical applications at all, and appeared at the same time as the Kalman filter and nearly 20 years before Reid’s seminal MHT paper [16]. Nearly a half-century after [15], FISST was devised as a novel application of *stochastic geometry* (not p.p. theory) to a specific engineering application: multitarget tracking and information fusion. It is this original application that requires proper attribution.

To state the issue plainly: In the *recent engineering tracking literature, the “p.p.” model of a random multitarget state* in Refs. [12,13,14] is, historically speaking, being promoted as an *alternative* to the *original FISST RFS model of a random multitarget state—not the other way around*.

### 2.2. RFS’s Are Simpler than Simple p.p.’s

Contrast the definition of a p.p. previously given with the definition of an RFS:

An RFS Ξ of the single-target state space ℑ is a random variable whose realizations are the finite subsets *X* = {**x**_1_, …, **x***_n_*} of ℑ of cardinality *n* ≥ 0.

This requires only simple concepts easily understood by engineers: random variable, finite set, cardinality. There is no need for measurable spaces, Borel sigma-algebras, or probability measures (symmetric or otherwise) (RFS’s do have a measure-theoretic basis, but in practical application it can usually be ignored—see Section 3.1.)

**Remark 1.**  *The fact that finite sets are order-free does not mean that we cannot distinguish between targets. In general, a single-target state will have the form **x** = (**u**, ℓ) where **u** is the kinematic state and ℓ is a uniquely identifying track label [1] (pp. 135, 196–197). This is the basis for the labeled RFS (LRFS) theory of Vo and Vo [7,8,9]; [3] (Chapter 15). In LRFS theory, **u** and ℓ are random variables and the ℓ are unordered symbols.*

### 2.3. Non-RFS p.p.’s Are Inappropriate for Multitarget Tracking

This is because *non-RFS p.p.’s are technically deficient representations of random multitarget states*. Every target track must have a unique identifying label—for example, “Bob.” Given this, **x** = (**u**, Bob) cannot occur more than once in (**x**_1_, …, **x***_n_*) since, otherwise, “Bob” would be present twice or more simultaneously. Thus *all state-p.p.’s must be simple*—i.e., *they must be RFS’s*—and so the claim that p.p.’s are “more general [than RFS’s]” is *false in actual engineering application*. And, in any case, immediately after this claim was made all p.p.’s were assumed to be simple.

### 2.4. Vectors Are Poor Multitarget State Representations

There are multiple reasons for this [17] (Section II-A); [18] (Section 4.2.3). (1) The targets in a multitarget population have no natural ordering. Imposing physically extraneous information on it, such as an order, risks the creation of unknown statistical biases. (2) If the system has n distinct targets *X* = {**x**_1_, …, **x***_n_*} then it has *n*! vector state representations *ϕ_X_*_,*π*_ = (**x***_π_*_1_, …, **x***_πn_*) (for permutations *π* on 1, …, *n*)—whereas ideally there should be a one-to-one correspondence between physical states and their representations. (3) The goal of multitarget algorithms is to produce estimates of the multitarget state that are as close as possible to ground truth. A *mathematical distance metric on multitarget states* is required to do so. Assume that there exists a metric *ρ*(*ϕ*_1_,*ϕ*_2_) on *vector states*. It must be constant under permutation of the entries of *ϕ*_1_ and *ϕ*_2_—in which case *ρ*(*ϕ*_1_,*ϕ*_2_) = *ρ*′(*χ*(*ϕ*_1_),*χ*(*ϕ*_2_)) for some *ρ*′, where *χ*(*ϕ*) denotes the set of entries in *ϕ*. Let *ϕ_π_* = (**x***_π_*_1_, …, x*_πn_*). Then *ρ*(*ϕ_π_*,*ϕ_π_*_′_) = *ρ*(*ϕ_π_*,*ϕ_π_*) = 0 for permutations *π* ≠ *π*′, contradicting the definition of a metric. That is: *no metric on vector states exists*. Finite sets have well-known metrics such as “OSPA” [3].

**Remark 2.**  *It could be argued that, because the probability distribution of a p.p. is symmetric, “…the order of points in a realization is irrelevant for statistical purposes” [13] (p. 1325). This is immaterial. Distance is an intrinsic, deterministic property of a multitarget state space that is independent of any particular probability distribution on that space.*

**Remark 3.**  *It should be pointed out that one of the authors of Refs. [12,13,14], as a coauthor of Ref. [18] (Section 4.2.3), marshaled similar arguments to similarly criticize vector representation.*

Finally, Ref. [13] appears to employ finite sets while the contrary is claimed. (1) Most obviously: “…the abuse of notation ‘*x* ∈ *ϕ*’ is used for ‘*x* ∈ *χ*(*ϕ*)’ where *χ* is the function associating a vector of distinct elements to the set composed of the same elements” [13] (footnote 3) (Such “abuse” is required only because vectors have been needlessly substituted in place of finite sets). (2) OSPA is used to measure distance between vectors (This is theoretically problematic since *ρ*(*ϕ*_1_,*ϕ*_2_) = *ρ*_OSPA_(*χ*(*ϕ*_1_),*χ*(*ϕ*_2_)) cannot be a metric). (3) The set-theoretic notation “Φ_1_ ∪ Φ_2_” is used to denote the “superposition” (i.e., set-theoretic union) of simple p.p.’s (i.e., RFS’s) Φ_1_, Φ_2_ [13] (Proposition 1).

### 2.5. Simple p.p.’s Produce a Flawed Mathematical Paraphrase of RFS’s

The replacement of every finite subset *X* = {**x**_1_, …, **x***_n_*} ⊆ ℑ with a vector *ϕ* = (**x**_1_, …, **x***_n_*) ∈ X and every RFS Ξ with a simple p.p. Φ results in a conceptually questionable and unnecessarily complexified mathematical paraphrase of FISST that does not correctly address the technical idiosyncrasies of the multitarget tracking application.

### 2.6. FISST is More General than MPMT

This is because FISST (a) has an integro-differential calculus of possibly nonadditive set functions and their density functions (Section 3.1); and (b) it Bayes-optimally addresses multitarget-multisource information fusion using “hard + soft” data in a unified manner [2] (Chapters 3–7); [3] (Chapter 22). The latter is attributable to the fact that FISST is based on stochastic geometry, which in turn is based on the theory of random closed subsets (RCS’s) [19], which in turn is the basis of FISST’s unification of “hard + soft” information fusion.

## 3. FISST Densities Replaced by “Measures”

MPMT replaces the former with the latter because:

“…a measure-theoretical formulation provides a more general framework that is required to construct certain statistical properties on point processes that can be exploited for practical applications; a recent example is given in [21] for the construction of the regional statistics…”.

[13] (p. 1325)

(The phrase “more general framework” is again logically vacuous: “more general” than what? What is implicitly meant is “more general than FISST”.) Here, “regional statistics” refers to the “regional variance” of Ref. [12]—i.e., the variance of the random integer |Ξ ∩ *S*|:

var_Ξ_(*S*) = E[|Ξ ∩ *S*|^2^] − E[|Ξ ∩ *S*|]^2^. (1)

In Ref. [12] it was claimed that the set function var_Ξ_(*S*) “…is… not a measure… [and so it] does not necessarily admit a density in general… This fact motivates the measure-theoretical approach…” This is not the case, because (as we shall see) var_Ξ_(*S*) *does admit a density.*

First, however, readers should be advised that the meaning of “measure” in Refs. [12,13,14] is often unclear. For example, since var_Ξ_(*S*) is *not* a measure, how can it motivate “the measure-theoretical approach”? Sometimes “measure” has its usual meaning: a nonnegative set function *μ*(*S*) such that *μ*(∪*_n_*_≥1_ *S_n_*) = ∑*_n_*_≥1_ *μ*(*S_n_*) for mutually disjoint *S_n_*. Other times, however, it means *nonadditive* set functions such as var_Ξ_(*S*).

The following subsections will address: the elements of FISST (Section 3.1); measure-theoretic p.p. theory (Section 3.2); the FISST density of the regional variance (Section 3.3); why measures are inappropriate for multitarget tracking (Section 3.4); and why the “measure-theoretical formulation” in Refs. [12,13,14] produces a complexified mathematical paraphrase of FISST (Section 3.5).

### 3.1. Basic Concepts of Finite-Set Statistics

This section is drawn from Ref. [4]. The theoretical basis of *single-target statistics* is the *probability measure p***_X_**(*S*) = Pr(**X** ∈ *S*) of a random vector **X** ∈ ℑ (not to be confused with the p.p. single-target state space **X** = ℑ). Single-target tracking requires the *probability density* of *p***_X_**(*S*):

(2) $$f_{X}(x)=\frac{dp_{X}}{d\lambda }(x)$$

where the right side is the Radon-Nikodým derivative of *p***_X_**(*S*) with respect to Lesbesgue measure *λ*(*S*) on ℑ ⊆ ℜ*^N^*. That is: *f***_X_**(**x**) has the property that ∫*_S_ f***_X_**(**x**)*d***x** = *p***_X_**(*S*) where ∫*_S_*·*d***x** = ∫⋅**1***_S_*(**x**)*dλ*(**x**) and **1***_S_*(**x**) is the indicator function of subset *S* ⊆ ℑ.

The goal of FISST was to *reformulate multitarget tracking as a generalized single-target tracking problem*, with RFS’s Ξ taking the place of random vectors **X**. The theoretical basis of *multitarget statistics* is the probability measure *p*_Ξ_(*O*) = Pr(Ξ ∈ *O*) over the Borel-measurable subsets *O* of the hyperspace whose elements are the finite subsets of single-target state space ℑ. (A “hyperspace” is a space whose elements are subsets of some other “base space.”) FISST avoids *p*_Ξ_(*O*) by equivalently replacing it with the stochastic-geometric *belief measure* (a.k.a. belief-mass function) *β*_Ξ_(*S*) = Pr(Ξ⊆*S*)—a conceptually simple generalization of *p***_X_**(*S*) = Pr(**X**∈*S*).

**Remark 4.**  *The belief measure can usually be avoided since it is usually necessary only for motion and measurement modeling—see Section 7.1*.

Multitarget tracking is based on the *multitarget probability density* of *β*_Ξ_(*S*)—i.e., the multitarget analog of Equation (2):

(3) $$f_{\Xi }(X)=\frac{\delta \beta_{\Xi }}{\delta X}(\emptyset )={\left[\frac{\delta \beta_{\Xi }}{\delta X}(S)\right]}_{S=\emptyset }$$

where Ø denotes the empty set and where the right side is a FISST *set derivative* of *β*_Ξ_(*S*) with respect to *X* = {**x**_1_, …, **x***_n_*} ⊆ ℑ. (The set derivative is a constructivist generalization of the Radon-Nikodým derivative: (*dp***_X_**/*dλ*)(**x**) = (*δp***_X_**/*δ*{**x**})(Ø)—see Ref. [4] (Section IV-F).)

A related density is the FISST multitarget factorial moment density:

(4) $$D_{\Xi }(X)=\frac{\delta \beta_{\Xi }}{\delta X}(ℑ)={\left[\frac{\delta \beta_{\Xi }}{\delta X}(S)\right]}_{S=ℑ}.$$

The special case *D*_Ξ_(**x**) = *D*_Ξ_({**x**}) is known as the *probability hypothesis density* (PHD) of Ξ.

**Remark 5.**  *f_Ξ_(X) and D_Ξ_(X) were defined in 1997 in Ref. [1] using stochastic geometry and set derivatives—not p.p. theory. Likewise for the first derivation [20] of the PHD filter.*

For any real-valued function *h*(**x**) and any finite *X* ⊆ ℑ, let *h^X^* = 1 if *X* = Ø and *h^X^* = ∏**_x_**_∈*X*_ *h*(**x**) otherwise. Then the multitarget analog of *p***_X_**(*S*) = ∫*_S_ f***_X_**(**x**)*d***x** is

(5) $$\beta_{\Xi }(S)=\int_{S}f_{\Xi }(X)\delta X=\int 1_{S}^{X}\cdot f_{\Xi }(X)\delta X,$$

where the *set integral* ∫ *f(X*)*δX* of a multitarget density function *f*(*X*) is defined as

(6) $$\int f(X)\delta X=\sum_{n\geq 0}\frac{1}{n!}\int f(\{x_{1},\ldots ,x_{n}\})dx_{1}\cdots dx_{n}.$$

The regional set integral ∫*_S_ f*(*X*)*δX* is nonadditive in *S* because *S* ↦ ∏**_x_**_∈*X*_ **1***_S_*(**x**) is nonadditive (It is not true that integrals must be additive in *S*—see, for example, [21].).

The set derivative has the following important property. Let *σ*(*S*) be a nonnegative set function defined on the closed subsets *S* ⊆ ℑ. Then if it exists, its FISST multitarget density is *σ*^∗^(*X*) = (*δσ*/*δX*)(Ø) since

(7) $$\int_{S}\frac{\delta \sigma }{\delta X}(\emptyset )\delta X=\sigma (S).$$

### 3.2. Measure-Theoretical p.p. Theory

The “measure-theoretical formulation” of p.p. theory in MPMT is stated as follows:

“The probability distribution *P*_Φ_ [of a simple p.p. Φ] is characterized by its projection measures *P*^(*n*)^_Φ_, for any *n* ≥ 0. The nth-order projection measure *P*^(*n*)^_Φ_, for any *n* ≥ 1, is defined on the Borel *σ*-algebra of **X***^n^* and gives the probability for the point process to be composed of *n* points, and the probability distribution of these points… For any *n* ≥ 0, *J*^(*n*)^_Φ_ denotes the *n*^th^-order Janossy measure…and is defined as *J*^(*n*)^_Φ_ (*B*_1_ × … × *B*_n_) = *n*!*P*^(*n*)^_Φ_(*B*_1_ × … × *B_n_*)… The probability density *p*_Φ_ (respectively (resp.) the *n*^th^-order projection density *p*^(*n*)^_Φ_, the *n*^th^-order Janossy density *j*^(*n*)^_Φ_) is the Radon-Nikodým derivative of the probability distribution *P*_Φ_ (resp. the *n*^th^-order projection measure *P*^(n)^_Φ_, the *n*^th^-order Janossy measure *J*^(*n*)^_Φ_) with respect to (w.r.t.) some reference measure… Throughout this article the exploitation of the Janossy measures will be preferred, for they are convenient tools in the context of functional differentiation…”.

[13] (p. 1325)

The “*k*th-order factorial moment measure” *M*^(*k*)^_Φ_(*B*_1_, …, *B_k_*) and its density *m*^(*k*)^_Φ_(*x*_1_, …, *x_k_*) are also introduced [13] (Equation (20)). MPMT is related to FISST as follows:

(8) $$j_{\Xi }^{\left(n\right)}(x_{1},\ldots ,x_{n})=\frac{1}{n!}\cdot f_{\Xi }(\{x_{1},\ldots ,x_{n}\})$$

(9) $$m_{\Xi }^{\left(n\right)}(x_{1},\ldots ,x_{n})=\frac{1}{n!}\cdot D_{\Xi }(\{x_{1},\ldots ,x_{n}\})$$

for distinct **x**_1_, …, **x***_n_*. (If **x**_1_, …, **x***_n_* are distinct then the factor 1/*n*! on the right sides of Equations (8) amd (9) apportions the probability of {**x**_1_, …, **x***_n_*} equally among the *n*! vectors that have the same elements as {**x**_1_, …, **x***_n_*}.). 

In summary: the families of multivariate measures *J*^(*k*)^_Φ_(*B*_1_, …, *B_k_*) resp. *M*^(*k*)^_Φ_(*B*_1_, …, *B_k_*) for *n* ≥ 1—i.e., the very measures that FISST rejected as unnecessary—have been substituted in place of *f*_Ξ_(*X*) resp. *D*_Ξ_(*X*).

This restoration—and thus MPMT—is allegedly unavoidable because (a) measures are “convenient tools” for “functional differentiation”; and (b) the fact that var_Ξ_(*S*) does not have a density proves that “…a measure-theoretical formulation provides a more general framework [than FISST]… for practical applications…” Neither assertion is true: var_Ξ_(*S*) does admit a density (Section 3.3); and measures are unnecessary for functional differentiation (Section 5.4).

Moreover, this restoration strips away a primary FISST insight: that all information about a multitarget system Ξ*_k_*_|*k*_ at time *t_k_* can be represented by a single multitarget probability density function *f_k_*_|*k*_(*X*|*Z*_1:*k*_)—i.e., the multitarget probability density function of Ξ*_k_*_|*k*_. The recent very fast implementations of the GLMB filter have been possible only because advanced stochastic sampling techniques can be applied to *f_k_*_|*k*_(*X*|*Z*_1:*k*_)—see Ref. [9] (pp. 1–2).

### 3.3. The FISST Multitarget Density of the Regional Variance

Contrary to the claim in Refs. [12,13,14], var_Ξ_(*S*) *does admit a density* even though it is not an additive measure. Specifically, recall the FISST set derivative (Section 3.1) and define:

(10) $${\mathrm{var}}_{\Xi }^{*}(X)=\frac{\delta {\mathrm{var}}_{\Xi }}{\delta X}(\emptyset ).$$

In Section 8.1 it is shown that this equals 0 unless |*X*| = 2, in which case

(11) $${\mathrm{var}}_{\Xi }^{*}(\{x_{1},x_{2}\})=2D_{\Xi }(\{x_{1},x_{2}\})+2\delta_{x_{1}}(x_{2})\cdot D_{\Xi }(x_{1})-2D_{\Xi }(x_{1})\cdot D_{\Xi }(x_{2})$$

for distinct **x**_1_, **x**_2_. By Equation (7) it must be the case that

(12) $$\int_{S}{\mathrm{var}}_{\Xi }^{*}(X)\delta X={\mathrm{var}}_{\Xi }(S).$$

This fact is verified in Section 8.2 for completeness. Thus var*_Ξ_ is the *FISST density* of var_Ξ_.

Consequently and contrary to claim, *the existence of the regional variance does not prove the unavoidability of MPMT*. Moreover, the fact that Equation (1) might be easier to use than Equation (11) in some circumstances is meager justification for wholesale adoption of formal measure theory (which in any case is inapplicable to var_Ξ_(*S*) since it is not an additive measure).

### 3.4. Measures Are Inappropriate for Practical Multitarget Tracking

This is because (a) *practical Bayes-optimal multitarget state estimation requires densities*; and (b) the multitarget Bayes’ rule as specified in Ref. [13] (Equation (22)),

(13) $$P_{k}(d\phi |Z_{1:k})=\frac{g_{k}(Z_{k}|\phi )\cdot P_{k|k-1}(d\phi |Z_{1:k-1})}{\int g_{k}(Z_{k}|\bar{\phi })\cdot P_{k|k-1}(d\bar{\phi }|Z_{k:k-1})},$$

requires the density function *P_k_*_|*k*−1_(*dϕ*|*Z*_1:*k*−1_) = *p_k_*_|*k*−1_(*ϕ*|*Z*_1:*k*−1_) in the numerator.

This point is implicitly conceded in [14] (p. 49), where the authors “…assume that… all [additive] measures studied in this article… admit densities”.

**Remark 6.**  *Indeed, P_k|k−1_(dϕ|Z_1:k−1_) is a mathematically equivalent substitution for the FISST f_k|k−1_(X|Z_1:k−1_); and ∫·P_k|k−1_(dϕ|Z_1:k−1_)= ∫·p_k|k−1_(ϕ|Z_1:k−1_)dλ_c_^∪^(ϕ) is an equivalent substitution for the FISST set integral ∫·f_k|k−1_(X|Z_1:k−1_)δX—see Section 4*.

**Remark 7.**  *It might nevertheless be objected that there is a purely measure-theoretic version of Bayes’ rule, the Killianpur-Striebel formula. It is immaterial since it is not employed in Refs. [12,13,14] despite the “measure-theoretical” emphasis of these papers. And if it had been, it would have only produced another mathematical paraphrase of FISST that begs the question: what significant engineering advances result from using it rather than Bayes’ rule?*

**Remark 8.**  *Since Dirac deltas are density functions, even singular measures can have density functions. For example, consider the bivariate measure μ_Ξ_(S_1_,S_2_) = E[|Ξ ∩ S_1_ ∩ S_2_|]. Its density function can be shown to be f(**x**,**y**) = δ**_y_**(**x**)·* *D_Ξ_(**y**). See also Equation (11).*

### 3.5. FISST Densities vs. Additive/Nonadditive Measures

For the purposes of multitarget tracking, families of multivariate measures, such as *J*^(*k*)^_Φ_(*B*_1_, …, *B_k_*) or *M*^(*k*)^_Φ_(*B*_1_, …, *B_k_*) for *n* ≥ 1, are mathematically equivalent to, but mathematically far more complicated than, the FISST multitarget density functions that they replace, such as *f*_Ξ_(*X*) and *D*_Ξ_(*X*). Consequently, replacing every FISST density with measures (or some other set function) produces a mathematically complexified mathematical paraphrase of FISST that is inappropriate for practical multitarget tracking *since densities are unavoidable*.

## 4. Set Integrals Replaced by Measure-Theoretic Integrals

The set integral ∫⋅*δX* was described in Section 3.1. MPMT replaces it with an integral ∫⋅*dλ*(*ϕ*) with respect to an *unspecified* “reference measure” *λ* [13] (Equation (2)). This is misleading, because *λ* cannot be arbitrary. If it is to be applicable to multitarget tracking it must be an extension of Lebesgue measure on ℑ ⊆ R*^N^* to ℑ^∞^ = ∪*_n_*_≥0_ ℑ*^n^*.

The following subsections address: the extension of Lesbesgue measure *λ* on ℑ ⊆ R*^N^* to a measure *λ_c_*^∪^ on ℑ^∞^ (Section 4.1); why the measure-theoretic integral ∫⋅*dλ_c_*^∪^(*ϕ*) is problematic from the point of view of practical multitarget tracking (Section 4.2); and why the substitution of ∫⋅*dλ_c_*^∪^(*ϕ*) in place of ∫⋅*δX* in Refs. [12,13,14] produces a conceptually flawed, complexified mathematical paraphrase of FISST (Section 4.3).

### 4.1. Extending Lesbegue Measure to Multitarget States

The following is drawn from Ref. [2] (Appendices F.3 and F.4). Suppose that ℑ ⊆ R*^N^* for some *N* and let *λ*(*S*) be Lesbesgue measure on ℑ. How can *λ* and Equation (2) be extended to ℑ^∞^ = ∪*_n_*_≥0_ ℑ*^n^*? Begin with *λ*. Let *λ^n^(O*′) be the usual extension of *λ* to the Cartesian-product space ℑ*^n^* for measurable *O*′ ⊆ ℑ*^n^*. Let *O* ⊆ ℑ^∞^ be measurable—i.e., *O*′^(*n*)^ = *O* ∩ ℑ*^n^* is measurable in ℑ*^n^* for every *n* ≥ 1, in which case *λ^n^*(*O*′^(*n*)^) exists for every *n* ≥ 1. If the unit of measurement in ℑ is *ι* then the unit of measurement of *λ^n^*(*O*′^(*n*)^) is *ι^n^*. Let *c* > 0 be a constant whose unit of measurement is *ι*. Define the extension of *λ* to ℑ^∞^ as:

(14) $$\lambda_{c}^{\cup }(O)=\sum_{n\geq 0}\frac{\lambda^{n}(O^{\left(n\right)})}{c^{n}}.$$

This is well-defined since each term in the sum is unitless.

Next let *f*(*ϕ*) be a unitless, nonnegative function of *ϕ* ∈ ℑ^∞^ and abbreviate *f*(**x**_1_, …, **x***_n_*) = *f*((**x**_1_, …, **x***_n_*)) and ∫⋅*d***x**_1_⋯*d***x***_n_* = ∫⋅*dλ^n^*(**x**_1_, …, **x***_n_*). Then it is integrable with respect to *λ_c_*^∪^ if the following exists:

(15) $$\int_{O}f(\phi )d\lambda_{c}^{\cup }(\phi )=\sum_{n\geq 0}\frac{1}{c^{n}}\int_{O^{\left(n\right)}}f(x_{1},\ldots ,x_{n})dx_{1}\cdots dx_{n}.$$

Now turn to the generalization of Equation (2). Let *μ*(*O*) be a probability measure on ℑ^∞^ and let *μ^n^* denote its restriction to ℑ*^n^*. Recall that *μ* is absolutely continuous with respect to (a.c.w.r.t.) another measure *μ*_0_ if *μ*(*O*) = 0 whenever *μ*_0_(*O*) = 0. If *μ* is a.c.w.r.t. *λ_c_*^∪^ then *μ^n^* is a.c.w.r.t. *λ^n^* for all *n* ≥ 1. Consequently, by the Radon-Nikodým theorem, for each *n* ≥ 1 there is an almost everywhere unique *f_n_*(*ϕ*) on *ϕ* ∈ ℑ*^n^* such that

(16) $$\mu^{n}(O^{\prime })=\int_{O^{\prime }}f_{n}(\phi )d\lambda^{n}(\phi )=\int_{O^{\prime }}f(x_{1},\ldots ,x_{n})dx_{1}\cdots dx_{n}$$

for all measurable *O*′ ⊆ ℑ*^n^*. The unit of measurement of *f_n_*(*ϕ*) is *ι*^−*n*^. Define the unitless function *f_c_*(*ϕ*) = *c^n^·f_n_*(*ϕ*) if *ϕ* = (**x**_1_, …, **x***_n_*). Then

(17) $$\mu (O)=\mu (\cup_{n>0}O^{\left(n\right)})=\sum_{n\geq 0}\mu^{n}(O^{\left(n\right)})=\sum_{n\geq 0}\int_{O^{\left(n\right)}}f_{n}(x_{1},\ldots ,x_{n})dx_{1}\cdots dx_{n}$$

(18) $$=\sum_{n\geq 0}\frac{1}{c^{n}}\int_{O^{\left(n\right)}}f_{c}(x_{1},\ldots ,x_{n})dx_{1}\cdots dx_{n}=\int_{O}f_{c}(\phi )d\lambda_{c}^{\cup }(\phi )$$

for all measurable *O* ⊆ ℑ^∞^. That is: *f_c_*(*ϕ*) = (*dμ*/*dλ_c_*^∪^)(*ϕ*) is the Radon-Nikodým density of *μ*(*O*) w.r.t. *λ_c_*^∪^—i.e., it is the extension of Equation (2) to ℑ^∞^ (If *μ* = *P*_Φ_ it is what in Ref. [13] is denoted as *P*_Φ_(*dϕ*) or *p*_Φ_(*ϕ*)).

This is *conceptually troublesome* since *μ* has a different density for each *c* > 0. In p.p. theory, the usual resolution of this difficulty is to set *c* = 1⋅*ι* [22] (pp. 1226–1229). But as we shall now see, this leads to a new conceptual difficulty when applied to multitarget tracking.

### 4.2. Measure-Theoretic Integrals and Multitarget State Estimation

Define the FISST multitarget density function *f*(*X*) by

*f(*{**x**_1_, …, **x***_n_*}) = *n*!·*f_n_*(**x**_1_, …, **x***_n_*) (19)

for distinct **x**_1_, …, **x***_n_*. From Equations (6), (18) and (20), the measure-theoretic and set integrals are equivalent:

(20) $$\int_{}f_{c}(\phi )d\lambda_{c}^{\cup }(\phi )=\int f(X)\delta X.$$

Also, the maximum a posteriori estimate

*ϕ_c_* = argsup*_ϕ_ f_c_*(*ϕ*) (21)

of *f_c_*(*ϕ*) is equivalent to FISST’s JoM (Joint Multitarget) estimate of *f*(*X*) [2] (p. 498):

(22) $$X_{c}=\arg\sup_{X}.\frac{c^{\left|X\right|}}{|X|!}\cdot f(X).$$

As was explained in Ref. [2] (pp. 499–500), to arrive at an intuitively reasonable *X_c_* the magnitude of *c* should be approximately equal to the accuracy with which any **x** ∈ ℑ can be estimated. Since this argument is fairly lengthy and involved, it cannot be reproduced here.

The *fixed* choice *c* = 1⋅*ι* will, in general, produce poor JoM estimates of *f(X*) (and therefore poor MAP estimates of *f_c_*(*ϕ*)). The only reasonable resolution is to attach *c* to a particular estimator—JoM—rather than to so fundamental a concept as a multitarget integral.

### 4.3. Set Integrals vs. Measure-Theoretic Integrals

The measure-theoretic integral ∫⋅*dλ_c_*^∪^(*ϕ*) is mathematically equivalent to but mathematically far more complicated than the set integral ∫⋅*δX*, which is not measure-theoretic. Also, from the point of view of practical multitarget tracking ∫⋅*δX* resp. *f*(*X*) resp. *X_c_* are preferable to ∫⋅*dλ_c_*^∪^(*ϕ*) resp. *f_c_*(*ϕ*) = *P*_Φ_(*dϕ*) resp. *ϕ_c_*. Consequently, replacing every set integral with a measure-theoretic integral, and every multitarget density with a Radon-Nikodým derivative, produces a flawed, complexified mathematical paraphrase of FISST.

## 5. Functional Derivatives Replaced by “Chain Differentials”

In MPMT the former is replaced with the latter “…so that a general chain rule can be determined…” [13] (p. 1326). The *plain meaning* of this phrase is: *the chain differential is necessary for a general chain rule (as applied in Ref.* [13] *to p.g.fl.’s).* It is false for two reasons:

1. The FISST functional derivative already has a general chain rule—see [23]; [3] (pp. 78–79).
2. When applied to p.g.fl.’s the chain differential is identical to the Gâteaux and Frechét derivatives—and thus mathematically equivalent to the FISST functional derivative.

The following subsections address the following topics: probability generating functionals (Section 5.1); differentiation theory (Section 5.2); differentiation of p.g.fl.’s (Section 5.3); equivalence of chain differentials and functional derivatives (Section 5.4); and the chain differential produces a complexified mathematical paraphrase of FISST (Section 5.5).

### 5.1. Probability Generating Functionals

The statistics of an RFS Ξ are equivalently characterized by *β*_Ξ_(*S*) and *f*_Ξ_(*X*). A third fundamental statistical descriptor of Ξ, the *probability generating functional* (p.g.fl.), is:

(23) $$G_{\Xi }[h]=\int h^{X}\cdot f_{\Xi }(X)\delta X$$

where the notation *h^X^* was defined in Equation (5). For present purposes the “test function” *h* will be assumed to be a nonnegative bounded function, in which case 0 ≤ *G*_Ξ_[*h*] < ∞. (FISST follows the practice in Ref. [24] of further assuming that 0 ≤ *h*(**x**) ≤ 1.) Note that *G*_Ξ_[**1***_S_*] = *β*_Ξ_(*S*).

A great many generating functionals besides the p.g.fl. are used in p.p. theory: characteristic, Laplace, moment, factorial-moment, cumulant, factorial-cumulant, Khinchin, etc., [24]. It was FISST that identified the particular importance of the p.g.fl. for multitarget tracking. 

The p.g.fl. finds its greatest use in the derivation of approximate multitarget filters such as the PHD and cardinalized PHD (CPHD) filters. This, in turn, requires a differential calculus of p.g.fl.’s—the subject of the next two subsections.

### 5.2. Differentiation Theory

Let *A*, *B* be (possibly infinite-dimensional) topological linear spaces and let *τ*: *A* → *B* be a transformation. Then the *Gâteaux differential* is a simple and obvious generalization of the differential quotient of undergraduate calculus:

(24) $$(\delta \tau )(a^{\prime };a)=\underset{\varepsilon \to 0}{\lim}\frac{\tau (a^{\prime }+\varepsilon \cdot a)-\tau (a^{\prime })}{\varepsilon }.$$

If the function defined by *a* ↦ (*δτ*)(*a*′;*a*) exists and is linear and continuous then (*δτ*)(*a*′;⋅) is called the Gâteaux *derivative* of *τ* at *a*′.

Now recall that a Banach space is a normed topological linear space that is closed with respect to limits. (A norm is a nonnegative function ‖**x**‖ such that ‖**x**‖ = 0 implies **x** = 0 and which satisfies the triangle inequality: ‖**x**+**y**‖ ≤ ‖**x**‖+‖**y**‖.)

Let *A*, *B* be Banach spaces with respective norms ‖⋅‖*_A_* and ‖⋅‖*_B_*. If there exists a linear-continuous function *D_a_*_′_*τ*: *A* → *B* such that

(25) $$\underset{a\to 0}{\lim}\frac{{\left\|\tau (a^{\prime }+a)-\tau (a^{\prime })-(D_{a{}^{\prime }}\tau )(a)\right\|}_{B}}{{\left\|a\right\|}_{A}}=0$$

then *D_a_*_′_*τ* is called the *Frechét derivative* of *τ* at *a*′. If the Frechét derivative exists then so does the Gâteaux derivative, and the two are equal.

The Frechét derivative admits a chain rule in the following sense. Let *ψ*: *B* → *C* be a second transformation between Banach spaces. If the Frechét derivatives of *τ* and *ψ* exist at *a*′ resp. *τ* (*a*′) then so does the Frechét derivative of (*τ*∘*ψ*)(*a*) = *ψ*(*τ* (*a*)) at *a*′ and it is:

(*D_a_*_′_(*τ*∘*ψ*))(*a*) = (*D_τ_*_(*a*′)_*ψ*)((*D_a_*_′_*τ*)(*a*)). (26)

Because the Gâteaux differential does not admit a chain rule in general, Bernard [25] devised a restricted version of it that does: the “chain differential.” It is defined as

(27) $$(\delta^{*}\tau )(a^{\prime };a)=\underset{n\to \infty }{\lim}\frac{\tau (a^{\prime }+\varepsilon_{n}\cdot a_{n})-\tau (a^{\prime })}{\varepsilon_{n}}$$

if the limit exists and is identical for any *ε_n_* → 0 and *a_n_* → *a*. If the chain differential exists then it is the Gâteaux differential [25] (Proposition 1). If *a* ↦ (δ*τ)(*a*′;*a*) exists and is linear and continuous then (δ*τ)(*a*′;⋅) is called the chain *derivative* of *τ* at *a*′ [25] (Proposition 1). If the Frechét derivative exists then it is equal to the chain derivative [25] (Proposition 1).

### 5.3. Differentiation of p.g.fl.’s

The Gâteaux and chain differentials of a p.g.fl. *G*_Ξ_[*h*] will be notated as, respectively,

(28) $$\frac{\partial G_{\Xi }}{\partial g}[h]=\underset{\varepsilon ↓0}{\lim}\frac{G_{\Xi }[h+\varepsilon \cdot g]-G_{\Xi }[h]}{\varepsilon }$$

(29) $$\frac{\partial^{*}G_{\Xi }}{\partial^{*}g}[h]=\underset{n\to \infty }{\lim}\frac{G_{\Xi }[h+\varepsilon_{n}\cdot g_{n}]-G_{\Xi }[h]}{\varepsilon_{n}}.$$

Suppose that *G*_Ξ_[*h*] is Gâteaux differentiable. Since *g*(**y**) = ∫*g*(**x**)⋅*δ***_x_**(**y**)*d***x** and *g* ↦ (*∂G*_Ξ_/*∂g*)[*h*] is linear and continuous it follows that, intuitively speaking,

(30) $$\frac{\partial G_{\Xi }}{\partial g}[h]=\int g(x)\cdot \frac{\partial G_{\Xi }}{\partial \delta_{x}}[h]dx$$

for all *g*. If it exists, the quantity

(31) $$\frac{\delta G_{\Xi }}{\delta x}[h]=\frac{\partial G_{\Xi }}{\partial \delta_{x}}[h]$$

is Volterra’s *functional derivative* of *G*_Ξ_[*h*] at **x** [26] (p. 75; p. 24, Equation (3)). Its significance is that it *permits the direct derivation of density functions without resort to measures* (and for this reason is preferred by the physics community [27,28]). Equation (30) shows that *the functional derivative is mathematically equivalent to the Gâteaux derivative*.

If *X* = {**x**_1_, …, **x***_n_*} with |*X*| = *n* then the *iterated functional derivative* is

(32) $$\frac{\delta G_{\Xi }}{\delta X}[h]=\frac{\delta^{n}G_{\Xi }}{\delta x_{1}\cdots \delta x_{n}}[h].$$

The set and functional derivatives are related by

(33) $$\frac{\delta \sigma }{\delta X}(S)=\frac{\delta \sigma^{+}}{\delta X}[1_{S}]$$

where *σ*^+^ is the p.g.fl. of σ*(*X*) = (*δσ*/*δX*)(Ø):

(34) $$\sigma^{+}[h]=\int h^{X}\cdot \frac{\delta \sigma }{\delta X}(\emptyset )\delta X.$$

Thus if *σ* = *β*_Ξ_ then:

(35) $$f_{\Xi }(X)=\frac{\delta G_{\Xi }}{\delta X}[0]=\frac{\delta \beta_{\Xi }}{\delta X}(\emptyset ).$$

In MPMT the space of test functions *h* is assumed to have the *L*_∞_ norm ‖*h*‖_∞_ = sup**_x_**_∈__ℑ_ |*h*(**x**)|—see Ref. [13] (p. 1326, footnote 2). The chain differential is therefore *superfluous* if the Frechét derivative of *G*_Ξ_[*h*] with respect to ‖·‖_∞_ exists. If so, it is given by:

(36) $$\underset{g↓0}{\lim}\frac{\left|G_{\Xi }[h+g]-G_{\Xi }[h]-(D_{h}G_{\Xi })[g]\right|}{{\left\|g\right\|}_{\infty }}=0.$$

### 5.4. Equivalence of Chain Differentials and Functional Derivatives of p.g.fl.’s

Let *G*_Ξ_[*h*] be the p.g.fl. of the RFS Ξ. Since (*h* + *εg*)*^X^* = ∑*_W_*_⊆_*_X_ h^X^*^−*W*^⋅*ε*^|*W*|^ *g^W^* (see Ref. [3] Equation (3.6)), Equation (28) becomes

(37) $$\frac{\partial G_{\Xi }}{\partial g}[h]=\int \left(\sum_{x\in X}h^{X-\{x\}}g(x)\right)\cdot f_{\Xi }(X)\delta X$$

(see Section 8.3). This is a Gâteaux *derivative* since it is linear and continuous in *g*. Equation (37) can be rewritten as

(38) $$\frac{\partial G_{\Xi }}{\partial g}[h]=\int g(x)\cdot \left(\int h^{X}\cdot f_{\Xi }(\{x\}\cup X)\delta X\right)dx$$

(see Section 8.4). From Equations (30) and (31), the quantity in the parentheses is the functional derivative:

(39) $$\frac{\delta G_{\Xi }}{\delta x}[h]=\int h^{X}\cdot f_{\Xi }(\{x\}\cup X)\delta X.$$

That is: the Gâteaux and functional derivatives of a p.g.fl. always exist. In Section 8.6 it is additionally shown that the Frechét derivative of a p.g.fl. always exists and is identical to the Gâteaux derivative: (*D_h_G*_Ξ_)[*g*] = (*∂G*_Ξ_/*∂g*)[*h*].

As for the chain differential of a p.g.fl., it is easily shown (see Section 8.5) that it always exists and is identical to the Gâteaux (and therefore the Frechét) derivative:

(40) $$\frac{\partial^{*}G_{\Xi }}{\partial^{*}g}[h]=\frac{\partial G_{\Xi }}{\partial g}[h]=(D_{h}G_{\Xi })[g].$$

Thus, by Equation (30), the density of the authors’ measure *S* ↦ (∂*G*_Ξ_/∂**1***_S_*)[*h*] is the functional derivative. The following two points are therefore established:

1. The general chain rule for p.g.fl.’s is a consequence of the Frechét derivative, not the superfluous chain differential.
2. The general chain rule for chain derivatives is mathematically equivalent to the general chain rule for functional derivatives and thus produces nothing new.

Specifically, let *G*_Ξ_[*T*[*h*]] = *G*_Ψ_[*h*] for some RFS Ψ. Then the chain rule for functional derivatives is Ref. [2] (Equation (11.285)):

(41) $$\frac{\delta }{\delta x}G_{\Xi }[T[h]]=\frac{\partial G_{\Xi }}{\partial \left(\frac{\delta T}{\delta x}[h]\right)}[T[h]]=\int \frac{\delta T}{\delta x}[h](w)\cdot \frac{\delta G_{\Xi }}{\delta w}[T[h]]dw$$

and the general chain rule for the functional derivative is Ref. [23]; [3] (Equation (3.91)):

(42) $$\frac{\delta }{\delta X}G_{\Xi }[T[h]]=\sum_{P⊥X}\frac{\partial G_{\Xi }}{\partial^{W\in P}\left(\frac{\delta T}{\delta W}[h]\right)}[T[h]]$$

where the summation is taken over all partitions *P* of *X*.

### 5.5. Functional Derivative vs. Chain Differential

The Gâteaux differential of a p.g.fl., Equation (28), is a simple and obvious generalization of the differential quotient of elementary calculus. The chain differential of a p.g.fl. is more complicated and by no means obvious. It is also identical to the Gâteaux and Frechét derivatives and therefore equivalent to the FISST functional derivative. Consequently: replacing every functional derivative with a chain differential produces a mathematically complexified paraphrase of FISST.

## 6. FISST Product Rule Replaced by “Leibniz’ Rule”

*”Leibniz’ Rule”is mathematically equivalent to the FISST product rule* since the chain differential is equivalent to the functional derivative. That is, “Leibniz’ rule” [13] (Equation (14))

(43) $$\delta^{n}(F\cdot G)(h;{\left(\eta \right)}_{i=1}^{n})=\sum_{\pi \subseteq \{1,\ldots ,n\}}\delta^{\left|\pi \right|}F(h;{\left(\eta \right)}_{i\in \pi })\delta^{n-|\pi |}G(h:{\left(\eta \right)}_{i\in \pi^{c}})$$

is substituted in place of its equivalent, the FISST product rule [2] (Equation (11.271)); [3] (Equation (3.70)):

(44) $$\frac{\delta }{\delta X}\left(F[h]\cdot G[h]\right)=\sum_{W\subseteq X}\frac{\delta F}{\delta (X-W)}[h]\cdot \frac{\delta G}{\delta W}[h].$$

Note the relative conceptual simplicity of the latter compared to the former. There is no mention of the FISST general product rule [2] (Equation (11.274)); [3] (Equation (3.68)):

(45) $$\frac{\delta }{\delta X}\left(F_{1}[h]\cdots F_{n}h]\right)=\sum_{W_{1}\dot{\cup }\ldots \dot{\cup }W_{n}=X}\frac{\delta F_{1}}{\delta W_{1}}[h]\cdots \frac{\delta F_{n}}{\delta W_{n}}[h].$$

More generally, there is no mention of the extensive FISST “toolbox” of general multitarget differentiation and integration rules [2] (pp. 383–389); [3] (pp. 69–80). MPMT instead replaces it with a paraphrase consisting of chain-differential rules that, for the purposes of multitarget tracking, are mathematically equivalent to their FISST counterparts [29]. For example, the formula [13] (Equation (16))

(46) $$\delta^{k}G_{\Phi }(h;\eta_{1},\ldots ,\eta_{k})=\sum_{n\geq k}\frac{1}{(n-k)!}\int \prod_{i=1}^{k}\eta_{i}(x_{i})\prod_{i=k+1}^{n}h(x_{i})J_{\Phi }^{\left(n\right)}(dx_{1},\ldots ,x_{n})$$

is substituted in place of, and is equivalent to, the decade-old FISST “generalized Radon-Nikodým” formula [2] (Equation (11.251)); [3] (pp. 69–80, 95–97)):

(47) $$\frac{\delta G_{\Xi }}{\delta X}[h]=\int h^{W}\cdot f_{\Xi }(X\cup W)\delta W.$$

Note the conceptual simplicity of the latter compared to the former.

**Remark 9.**  *Note that Equation (39) is the special case of Equation (47) with X = {**x**}.*

**Remark 10.**  *The paper [14] does contain three acknowledgements of the FISST “toolbox”: the FISST “generalized product rule for set derivatives” [14] (p. 51), the FISST “multi-target Bayesian recursion” [14] (p. 49), and the FISST “extraction rule…for the evaluation of the multitarget density of a RFS” [14] (p. 51). The paper [23] (in which the general chain rule for the FISST functional derivative is derived) is acknowledged [14] (p. 50)—but is cited as a chain differential paper even though it addresses only the functional derivative. These negligible differences aside, the issues raised in this paper apply with full force to [14] (not just [12,13])*.

## 7. RFS Motion Models Replaced by MPMT Motion Models

The paper [13] is devoted to a CPHD filter with target spawning, which in turn requires the predicted p.g.fl. *G_k_*_|*k*−1_[*h*]. This formula was derived 14 years earlier in Ref. [30] (p. 1173). An alleged p.p derivation of it is substituted in its place.

The following subsections address: the FISST multitarget motion model (Section 7.1); the FISST predicted p.g.fl. (Section 7.2); the FISST and p.p. multitarget motion models are identical (Section 7.3); and the FISST and p.p. predicted p.g.fl.’s are identical (Section 7.4).

### 7.1. The “Standard” FISST Multitarget Motion Model

This section is drawn from Sections III-C and IV-D of Ref. [4]. Single-target tracking is based on an explicit motion model. It typically has the form **X***_k_*_|*k*−1_ = *h_k_*_|*k*−1_(**x**′) + **W***_k_*_|*k*−1_ where **X***_k_*_|*k*−1_ is the random predicted target state, *h_k_*_|*k*−1_(**x**′) is the deterministic predicted state given that the target has state **x**′ at time *t_k_*_−1_, and **W***_k_*_|*k*−1_ is the plant noise. The statistics of this model are characterized by the probability measure *p_k_*_|*k*−1_(*S*|**x**′) = Pr(**X***_k_*_|*k*−1_ ∈ *S*|**X***_k−_*_1|*k*−1_ = **x**′). The Markov density *f_k_*_|*k*−1_(**x**|**x**′) is derived from it via calculus.

A fundamental innovation of FISST was to extend this reasoning to multitarget systems. Assume that there is no target spawning—i.e., a target at time *t_k_* survives or disappears but does not generate new targets. This scenario is described by the *RFS “standard” motion model*

(48) $$\Xi_{k|k-1}=T_{k|k-1}(x_{1}^{\prime })\cup \ldots \cup T_{k|k-1}(x_{n}^{\prime })\cup B_{k|k-1}.$$

Here, *X*′ = {**x**′_1_, …, **x**′*_n_*} with |*X*′| = *n* is the multitarget state at time *t_k_*_−1_; the RFS *T_k_*_|*k*−1_(**x**′) describes the evolution of a target with state **x**′; and the RFS *B_k_*_|*k*−1_ describes the newly-appearing targets. Here, either *T_k_*_|*k*−1_(**x**′) = ∅ (target vanishes with probability 1 − *p_S_*(**x**′)) or *T_k_*_|*k*−1_(**x**′) = {**X***_k_*_|*k*−1_} (target survives with probability *p_S_*(**x**′)). Also, *B_k_*_|*k*−1_ is assumed to be a Poisson RFS.

If targets evolve independently then the belief measure of Ξ*_k_*_|*k*−1_ factorizes as:

(49) $$\beta_{\Xi_{k|k-1}}(S|X{}^{\prime })=\beta_{T_{k|k-1}(x_{1}^{\prime })}(S)\cdots \beta_{T_{k|k-1}(x_{n}^{\prime })}(S)\cdot \beta_{B_{k|k-1}}(S).$$

Given this, Equation (35) and the FISST multitarget calculus rules are used to derive an explicit formula for the multitarget Markov density *f_k_*_|*k*−1_(*X*|*X*′) [2] (Equations (13.6)–(13.8)).

If there is target spawning, then *T_k_*_|*k*−1_(**x**′) is replaced by *T_k_*_|*k*−1_(**x**′) ∪ *T*′ *_k|k_*_−1_(**x**′) where the RFS *T*′*_k_*_|*k*−1_(**x**′) models the targets spawned by a target with state **x**′ at time *t_k_*_−1_.

### 7.2. The FISST Predicted p.g.fl.

Given the formula for *f_k_*_|*k*−1_(*X*|*X*′), one can derive the formula for the p.g.fl. *G_k_*_|*k*−1_[*h*] (of the predicted multitarget distribution *f_k_*_|*k*−1_(*X*|*Z*_1:*k*−1_)) in terms of the p.g.fl. *G_k−_*_1|*k*−1_[*h*] of the prior distribution *f_k−_*_1|*k*−1_(*X*|*Z*_1:*k*−1_), as follows [2] (Equation (16.399)); [3] (p. 1167):

*G_k_*_|*k*−1_[*h*] = *e_h_*·*G_k−_*_1|*k*−1_[(1 − *p_S_*+ *p_S_ p_h_*)⋅*b_h_*]. (50)

Here, *p_h_*(**x**′) = ∫ *h*(**x**)⋅*f_k_*_|*k*−1_(**x**|**x**′)*d***x** describes the surviving targets; *b_h_*(**x**′) = ∫ *h^X^*⋅*b_k_*_|*k*−1_(*X*|**x**′)*δX* describes the spawned targets; and *e_h_*{*h*} = ∫ *h^X^*⋅*b_k_*_|*k*−1_(*X*)*δX* describes the appearing targets.

### 7.3. The FISST and MPMT Motion Models Are Identical

In Ref. [13] the RFS motion model is implicitly presumed and a “p.p.” paraphrase of it substituted in its place. Specifically, *T_k_*_|*k*−1_(**x**′) is replaced by a surviving “daughter” p.p. Φ*_s_*; *T*′*_k_*_|*k*−1_(**x**′) is replaced by a “spawning point process” Φ*_b_*; *B_k_*_|*k*−1_ is replaced by a “spontaneous birth process” Φ*_γ_*; and Ξ*_k_*_|*k*−1_ is replaced by the “predicted multitarget process” Φ*_k_*_|*k*−1_.

### 7.4. The FISST and MPMT Predicted p.g.fl.’s Are Identical

In Ref. [13] (Equation (62b)) the “Galton-Watson equation” and other formulas are used to derive the predicted p.g.fl.:

*G_k_*_|*k*−1_[*h*] = *G_γ_*[*h*]·*G_k−1_*_|*k*−1_[G*_s_*[*h*|⋅]⋅*G_b_*[*h*|⋅]] (51)

where *G_γ_*[*h*] is identical to *e_h_*; *G_s_*[*h*|⋅] is identical to 1 − *p_S_*(⋅) + *p_S_*((⋅)*p_h_*(⋅); and *G_b_*[*h*|⋅] is identical to *b_h_*. That is: Equation (51) is the result of a “p.p.” derivation that is nearly identical to the FISST derivation, and is exactly the same formula that was derived using FISST 14 years earlier.

## 8. Mathematical Derivations

The theoretical results reported in this section are original. Even so, the results reported in Section 8.3, Section 8.4, Section 8.5 and Section 8.6—i.e., the existence and equality of the Frechét, Gâteaux, and chain derivatives of a p.g.fl.—should be regarded, from an intuitive point of view, as nearly obvious. A p.g.fl. *G*[*h*] is a functional analog of a power-series function *f*(*x*) = ∑*_n_*_≥0_ *a_n_x^n^*. (Indeed, it is an instance of what Volterra in Ref. [26] called a “functional power series.”) Since a power-series function is analytic—i.e., its Newtonian derivatives (*d^n^f*/*dx^n^*)(*x*) of arbitrary order *n* exist, with (*d^n^f*/*dx^n^*)(0) = *n*!·*a_n_*—it should not be surprising that p.g.fl.’s are analogously analytic.

### 8.1. Derivation of the Density Function of the Regional Variance

We are to prove Equation (11). First extend var_Ξ_(*S*) to a functional as follows:

(52) $${\mathrm{var}}_{\Xi }^{+}[h]=\int {\left(\sum_{x\in X}^{}h(x)\right)}^{2}\cdot f_{\Xi }(X)\delta X-{\left(\int \left(\sum_{x\in X}^{}h(x)\right)\cdot f_{\Xi }(X)\delta X\right)}^{2}.$$

It is easily seen that var_Ξ_(*S*) = var^+^_Ξ_[**1***_S_*]. Thus, from Equation (33) we get:

(53) $${\mathrm{var}}_{\Xi }^{*}(S)=\frac{\delta {\mathrm{var}}_{\Xi }}{\delta X}(\emptyset )=\frac{\delta {\mathrm{var}}_{\Xi }^{+}}{\delta X}[0].$$

By Campbell’s theorem [3] (Equation (4.96)), the second term of Equation (52) can be simplified:

(54) $${\mathrm{var}}_{\Xi }^{+}[h]=\int {\left(\sum_{x\in X}^{}h(x)\right)}^{2}\cdot f_{\Xi }(X)\delta X-{\left(\int h(x)\cdot D_{\Xi }(x)dx\right)}^{2}.$$

Taking functional derivatives *δ*/*δ***x**_1_ and *δ*/*δ***x**_2_ of Equation (56) with **x**_1_ ≠ **x**_2_ we get:

(55) $$\frac{\delta {\mathrm{var}}_{\Xi }^{+}}{\delta x_{1}}[h]=\int 2\left(\sum_{x\in X}^{}h(x)\right)\left(\sum_{x{}^{\prime }\in X}^{}\delta_{x_{1}}(x{}^{\prime })\right)\cdot f_{\Xi }(X)\delta X-2\left(\int h(x)\cdot D_{\Xi }(x)dx\right)\left(\int \delta_{x_{1}}(x)\cdot D_{\Xi }(x)dx\right)$$

(56) $$=\int 2\left(\sum_{x\in X}^{}h(x)\right)\left(\sum_{x{}^{\prime }\in X}^{}\delta_{x_{1}}(x{}^{\prime })\right)\cdot f_{\Xi }(X)\delta X-2D_{\Xi }(x_{1})\left(\int h(x)\cdot D_{\Xi }(x)dx\right)$$

and so

(57) $$\frac{\delta^{2}{\mathrm{var}}_{\Xi }^{+}}{\delta x_{2}\delta x_{1}}[h]=\int 2\left(\sum_{x\in X}^{}\delta_{x_{2}}(x)\right)\left(\sum_{x{}^{\prime }\in X}^{}\delta_{x_{1}}(x{}^{\prime })\right)\cdot f_{\Xi }(X)\delta X-2D_{\Xi }(x_{1})\cdot D_{\Xi }(x_{2}).$$

The quadratic version of Campbell’s theorem is [3] (Equation (4.102)):

(58) $$\int \left(\sum_{y\in Y}^{}h(y)\right)\left(\sum_{y{}^{\prime }\in Y}^{}h{}^{\prime }(y{}^{\prime })\right)\cdot f_{\Xi }(Y)\delta Y=\int h(y)\cdot h{}^{\prime }(y)\cdot D_{\Xi }(y)dy+\int h(y_{1})\cdot h{}^{\prime }(y_{2})\cdot D_{\Xi }(\{y_{1},y_{2}\})dy_{1}dy_{2}.$$

Given this and and since **x**_1_ ≠ **x**_2_,

(59) $$\int \left(\sum_{y\in Y}^{}\delta_{x_{1}}(y)\right)\left(\sum_{y{}^{\prime }\in Y}^{}\delta_{x_{2}}(y{}^{\prime })\right)\cdot f_{\Xi }(Y)\delta Y=\int \delta_{x_{1}}(y)\cdot \delta_{x_{2}}(y)\cdot D_{\Xi }(y)dy+\int \delta_{x_{1}}(y_{1})\cdot \delta_{x_{2}}(y_{2})\cdot D_{\Xi }(\{y_{1},y_{2}\})dy_{1}dy_{2}$$

(60) $$=\delta_{x_{1}}(x_{2})\cdot D_{\Xi }(x_{1})+D_{\Xi }(\{x_{1},x_{2}\}).$$

Thus after setting *h* = 0, Equation (57) yields Equation (11).

### 8.2. The Set Integral of the Reegional-Variance Density Is the Regional Variance

We are to prove Equation (12). From Equations (6) and (11) the set integral of var^+^_Ξ_(*X*) is

(61) $$\int_{S}{\mathrm{var}}_{\Xi }^{*}(X)\delta X=\int_{S\cdot S}D_{\Xi }(\{x_{1},x_{2}\})dx_{1}dx_{2}+\int_{S\cdot S}\delta_{x_{1}}(x_{2})\cdot D_{\Xi }(x_{1})dx_{1}dx_{2}-{\left(\int_{S}D_{\Xi }(x)dx\right)}^{2}$$

(62) $$=\int_{S\cdot S}D_{\Xi }(\{x_{1},x_{2}\})dx_{1}dx_{2}+\int_{S}D_{\Xi }(x)dx-E{\left[|\Xi \cap S|\right]}^{2}.$$

Setting *h* = *h*′ = **1***_S_* in Equation (58) results in

(63) $$\int_{S\cdot S}D_{\Xi }(\{y_{1},y_{2}\})dy_{1}dy_{2}=E[|\Xi \cap S{|}^{2}]-\int_{S}D_{\Xi }(y)dy.$$

Substituting this into Equation (62) we get, as claimed, var_Ξ_(*S*).

### 8.3. The Gâteau Differential of a p.g.fl.

We are to prove Equation (37). By Equation (28) the Gâteaux differential of *G*_Ξ_[*h*] is

(64) $$\frac{\partial G_{\Xi }}{\partial g}[h]=\underset{\varepsilon ↓0}{\lim}\frac{G_{\Xi }[h+\varepsilon \cdot g]-G_{\Xi }[h]}{\varepsilon }.$$

Since (*h* + *εg*)*^X^* = ∑*_W_*_⊆*X*_ *h^X^*^−*W*^⋅*ε*^|*W*|^ *g^W^* [3] (Equation (3.6))),

(65) $$G_{\Xi }[h+\varepsilon \cdot g]=\int \left(\sum_{W\subseteq X}h^{X-W}\cdot \varepsilon^{\left|W\right|}g^{W}\right)\cdot f_{\Xi }(X)\delta X$$

(66) $$=G_{\Xi }[h]+\varepsilon \int \left(\sum_{x\in X}h^{X-\{x\}}g(x)\right)\cdot f_{\Xi }(X)\delta X+\varepsilon^{2}\int \left(\sum_{W\subseteq X,|W|\geq 2}h^{X-W}\cdot \varepsilon^{|W|-2}g^{W}\right)\cdot f_{\Xi }(X)\delta X.$$

From this Equation (37) immediately follows.

### 8.4. Formula for the Functional Derivative of a p.g.fl.

We are to prove Equation (38). From the definition of a set integral, Equation (6), we see that

(67) $$\begin{array}{ll} \int \left(\sum_{x\in X}h^{X-\{x\}}g(x)\right)\cdot f_{\Xi }(X)\delta X= & \sum_{n\geq 0}\frac{1}{n!}\int h(x_{2})\cdots h(x_{n})\cdot g(x_{1})\cdot f_{\Xi }(\{x_{1},\ldots ,x_{n}\})dx_{1}\cdots dx_{n}\\ & +\ldots \\ & \sum_{n\geq 0}\frac{1}{n!}\int h(x_{1})\cdots h(x_{n-1})\cdot g(x_{n})\cdot f_{\Xi }(\{x_{1},\ldots ,x_{n}\})dx_{1}\cdots dx_{n} \end{array}$$

(68) $$=\sum_{n\geq 0}\frac{n}{n!}\int g(x)\cdot h(x_{1})\cdots h(x_{n-1})\cdot f_{\Xi }(\{x,x_{1},\ldots ,x_{n-1}\})dxdx_{1}\cdots dx_{n-1}$$

(69) $$=\sum_{i\geq 0}\frac{1}{i!}\int g(x)\cdot h(x_{1})\cdots h(x_{i})\cdot f_{\Xi }(\{x,x_{1},\ldots ,x_{i}\})dxdx_{1}\cdots dx_{i}$$

(70) $$=\int g(x)\cdot \left(\int h^{X}\cdot f_{\Xi }(\{x\}\cup X)\right)\delta Xdx.$$

### 8.5. The Chain Differential of a p.g.fl.

We are to prove Equation (40). From Equations (65) and (66) and the definition of the chain differential, Equation (29),

(71) $$G_{\Xi }[h+\varepsilon_{n}g_{n}]=\int \left(\sum_{W\subseteq X}h^{X-W}\cdot \varepsilon_{n}^{\left|W\right|}g_{n}^{W}\right)\cdot f_{\Xi }(X)\delta X$$

(72) $$=G_{\Xi }[h]+\varepsilon_{n}\int \left(\sum_{x\in X}h^{X-\{x\}}g_{n}(x)\right)\cdot f_{\Xi }(X)\delta X+\varepsilon_{n}^{2}\int \left(\sum_{W\subseteq X,|W|\geq 2}h^{X-W}\cdot \varepsilon_{n}^{|W|-2}g_{n}^{W}\right)\cdot f_{\Xi }(X)\delta X.$$

Equation (40) immediately follows from this.

### 8.6. The Frechét Derivative of a p.g.fl.

We are to show that, with respect to the *L*_∞_ norm ‖*h*‖_∞_ = sup**_x_**_∈_*_X_*|*h*(**x**)|, the Frechét derivative of a p.g.fl. exists. We know that if it exists then it must be equal to the Gâteaux derivative, which by Equation (37) is

(73) $$l_{h}[g]=\int \left(\sum_{x\in X}h^{X-\{x\}}g(x)\right)\cdot f_{\Xi }(X)\delta X.$$

Because of Equation (36) we are to show that

(74) $$\underset{g↓0}{\lim}\frac{\left|G_{\Xi }[h+g]-G_{\Xi }[h]-l_{h}[g]\right|}{{\left\|g\right\|}_{\infty }}=0.$$

However, the left side is easily seen to be

(75) $$\underset{g↓0}{\lim}\frac{\left|\int \sum_{W\subseteq X,|W|\geq 2}h^{X-W}g^{W}\cdot f_{\Xi }(x)\delta X\right|}{{\left\|g\right\|}_{\infty }}=\underset{g↓0}{\lim}\left|\int \sum_{W\subseteq X,|W|\geq 2}h^{X-W}\cdot \left(\frac{g^{W}}{\sup_{x}g(x)}\right)\cdot f_{\Xi }(x)\delta X\right|$$

(76) $$=\left|\int \sum_{W\subseteq X,|W|\geq 2}h^{X-W}\cdot \underset{g↓0}{\lim}\left(\frac{g^{W}}{\sup_{x}g(x)}\right)\cdot f_{\Xi }(x)\delta X\right|.$$

For fixed *W* = {**x**_1_, …, **x***_n_*} with |*W*| = *n* ≥ 2, the limit in Equation (76) is

(77) $$\underset{g↓0}{\lim}\left(\frac{g(x_{1})\cdots g(x_{n})}{\sup_{x}g(x)}\right)\leq \underset{g↓0}{\lim}g(x_{1})\cdots g(x_{n-1})=0.$$

## 9. Conclusions

The finite-set statistics (FISST) approach to multitarget tracking—stochastic geometry, random finite sets (RFS’s), belief-mass functions, and set derivatives—was introduced in the mid-1990s [1]. Its extended form—probability generating functionals (p.g.fl.’s) and Volterra functional derivatives [2,3,4]—dates from 2001 [5]. An allegedly more general alternative to FISST—herein called “point-process measure-theoretical multitarget tracking” or “MPMT”—has been presented in Refs. [12,13,14]. Herein it was demonstrated that MPMT is a mathematical paraphrase of part of FISST with the following mathematically equivalent substitutions:

Finite set ⇒ vector; RFS ⇒ simple point process (p.p.); multitarget densities ⇒ “measures” (actually, set functions or families of measures); multitarget probability density ⇒ family of Janossy measures; multitarget factorial-moment density ⇒ family of factorial-moment measures; multitarget distribution *f_k_*_|*k*_(*X*|*Z*_1:*k*_) ⇒ Radon-Nikodým density *P_k_*_|*k*_(*dϕ*|*Z*_1:*k*_); set integral ⇒ measure-theoretic integral; functional derivative ⇒ chain differential; functional-derivative product rule ⇒ chain-differential “Leibniz’ rule”; functional-derivative chain rules ⇒ chain-derivative chain rules; RFS multitarget motion model ⇒ “p.p.” multitarget motion model; FISST predicted p.g.fl. ⇒ “p.p.” predicted p.g.fl.; and so on.

It was further demonstrated that each of these substitutions is an unnecessary mathematical complexification of the FISST component that it replaces. In particular:

- Vector multitarget-state representation is a mathematically equivalent complexification of finite set representation that is inappropriate for practical multitarget tracking.
- A simple p.p. is a mathematically equivalent complexification of an RFS that is inappropriate for practical multitarget tracking.
- The “regional variance” of Ref. [12] *does* admit a density—thereby refuting the only evidence offered in Refs. [12,13,14] that MPMT is unavoidable for practical multitarget tracking.
- The measure-theoretic integral is a mathematically equivalent complexification of the FISST set integral that is inappropriate for practical multitarget tracking.
- When applied to practical multitarget tracking, the “chain differential” is a mathematically equivalent complexification of the FISST functional derivative.

Beyond this, FISST is significantly more general than MPMT because it: (a) has an integro-differential calculus of nonadditive set functions and their densities; and (b) provides a provably Bayes-optimal unification of “hard + soft” multitarget information fusion.
